# Risk Factors for Non-arteritic Anterior Ischemic Optic Neuropathy: A Large Scale Meta-Analysis

**DOI:** 10.3389/fmed.2021.618353

**Published:** 2021-10-04

**Authors:** Bing Liu, Ying Yu, Wen Liu, Tuo Deng, Daoman Xiang

**Affiliations:** ^1^Department of Ophthalmology, Guangzhou Women and Children's Medical Center, Guangzhou Medical University, Guangzhou, China; ^2^Department of Urology and Guangdong Key Laboratory of Urology, The First Affiliated Hospital of Guangzhou Medical University, Guangzhou Medical University, Guangzhou, China

**Keywords:** risk factor, non-arteritic anterior ischemic optic neuropathy, large scale, meta-analysis, systematic review

## Abstract

**Objective:** We conducted a meta-analysis to explore all the potential risk factors for non-arteritic anterior ischemic optic neuropathy (NAION) based on the published literature.

**Methods:** A comprehensive literature search through the online databases was performed to obtain studies concerning the risk factors of NAION up to June 2020. Pooled unadjusted odds ratios (*OR*s) or rate ratios (*RR*s) were calculated to evaluate the weight of risk factors. This study was registered in PROSPERO under the number CRD42018084960.

**Results:** Our meta-analysis included 49 original studies comprising of more than 10 million patients. The following risk factors were proved to be significantly associated with NAION: male gender (*OR* = 1.67, 95% *CI*: 1.50–1.85, *P* < 0.00001), hypertension (*RR* = 1.28, 95% *CI*: 1.20–1.37, *P* < 0.00001), hyperlipidemia (*RR* = 1.43, 95% *CI*: 1.26–1.62, *P* < 0.00001), diabetes mellitus (DM) (*RR* = 1.53, 95% *CI*: 1.36–1.73, *P* < 0.00001), coronary heart disease (CHD) (*RR* = 1.68, 95% *CI*: 1.24–2.27, *P* = 0.0008), sleep apnea (*RR* = 3.28, 95% *CI*: 2.08–5.17, *P* < 0.00001), factor V Leiden heterozygous (*RR* = 2.21, 95% *CI*: 1.19–4.09, *P* = 0.01), and medication history of cardiovascular drugs.

**Conclusion:** We concluded that the above risk factors were significantly related to NAION. Better understanding of these risk factors in NAION can help the direct therapeutic approaches.

## Introduction

Non-arteritic anterior ischemic optic neuropathy (NAION) is the most common ischemic optic neuropathy. The incidence rate is 2.5–11.8 per 100,000 cases in men elder than 50 (1). Characterized by optic nerve ischemia mostly due to hypoperfusion of short posterior ciliary arteries (SPCAs) (2), NAION can lead to unilateral, sudden, and painless loss of vision among awake patients. Segmental or diffuse optic disc edema can be observed without evidence of arteritis (3). Although the detailed pathogenesis is unclear, NAION is probably related to systematic hypoperfusion, nocturnal hypotension, local autoregulation failure, and hypercoagulation (2). NAION is a naturally progressive disease and the contralateral eye involvement rate is 15–20% in the following 5 years (4). It has been proved that the medications, including corticosteroids, aspirin, and neurotrophic drugs, have limited and controversial efficacies (5, 6). The risk factors should be taken into thorough consideration when providing precautions and treatments for NAION.

The elder men were susceptible to NAION (1, 3). NAION-related cardiac and cerebral vascular diseases were reported: hypertension, diabetes mellitus (DM), hyperlipidemia, stroke, prothrombotic disorders, and so on (2). Other systematic and ocular factors were also researched in the previously published articles, such as smoking (1), obstructive sleep apnea (7), phosphodiesterase type-5 inhibitors (PDE5-Is) (8), depression, anemia (9), chronic obstructive pulmonary disease (COPD) (10), hypothyroidism (11), small optic disc (12), age-related macular degeneration, and glaucoma (9). Furthermore, several specific genetic polymorphisms were tested for their association with NAION, involving ACE I/D, MTHFR C677T, and factor V Leiden (13).

The two published meta-analyses reported influences of DM or sleep apnea on NAION, respectively (7, 14). However, impacts of other factors differed in literature and no cumulative conclusions were reached. Therefore, we decided to perform a large-scale systematic review and meta-analysis on all the possible NAION risk factors identified in the published studies. To the best of our knowledge, this is the first meta-analysis concentrating on multiple factors of NAION. We expect to help the clinicians comprehensively understand the risk factors of NAION and provide more evidence for the preventions and treatments.

## Methods

We conducted this systematic review and meta-analysis in accordance with the meta-analysis of observational studies in epidemiology (MOOSE) guidelines. The protocol registration number of PROSPERO (https://www.crd.york.ac.uk/prospero/) was CRD42018084960.

### Search Strategy and Study Selection

Original literature was searched comprehensively through electronic Pubmed, Medline, Embase, and Cochrane Library databases. The related references were also screened, including gray literature (in the website http://graylit.osti.gov/). The language of included studies was restricted to English. The last search was on June 6, 2020. The search terms were applied as follows: “non-arteritic anterior ischemic optic neuropathy” OR “non-arteritic anterior ischaemic optic neuropathy” OR “NAION” OR “NA-AION” in combination with “risk” OR “factor” OR “risk factor.” These terms were searched in all the fields of articles, not restricted to their abstracts.

The inclusion criteria were listed as follows. First, the clinical studies concerning the comparisons of risk factors between NAION and controls were taken into further consideration. Second, the risk factors should exist before the diagnosis of NAION, which was judged by carefully screening the abstracts and/or full texts. Third, the samples of case and control groups were provided directly, or odds ratios (*OR*s) or rate ratios (*RR*s) of risk factors were reported with 95% *CI*s. Accordingly, we excluded the animal experiments, case reports/series, abstracts, conference proceedings, repeated publications, non-published materials, reviews, and editorials.

Two investigators (B. L. and Y. Y.) did the literature search, study screening, data extraction, and eligible study quality assessment independently. The inconsistency was resolved by a third reviewer or *via* an open discussion.

### Data Extraction and Study Quality Assessment

We collected the following data in a prepared standard form: first author, year of publication, country, ethnicity, study design, study duration, sample size, baseline information of the patient, and the number of patients (*OR*s or *RR*s with 95% *CI*s) in both the NAION and the control groups. The Newcastle-Ottawa Scale (NOS) (15) was applied for quality assessment of the non-randomized controlled trials (RCTs). The studies achieving seven or more stars were regarded as high quality.

### Statistics Analysis

We calculated the pooled unadjusted *OR*s or *RR*s on dichotomous variables to identify the association between the risk factors and NAION. Accordingly, the mean differences (MDs) were used on the continuous variables. Heterogeneity was determined through the chi-square test based on *Q* and *I*^2^ values (16). No significant heterogeneity existed if the *p*-value was >0.10, and in this condition, the fixed-effect model was used. On the contrary, the random-effect model was applied. We also conducted the subgroup analyses according to different populations. The results were significant in our meta-analysis if a two-sided *p*-value was < 0.05. Inverted funnel plot visual inspection was to assess the publication bias for all the comparisons, and the Egger's test was added when the number of studies was more than 10. The data analyses were performed in software RevMan (version 5.3; Cochrane Collaboration, Oxford, UK) and STATA (version 13.0; StataCorp, College Station, TX, USA).

## Results

**Figure 1:**
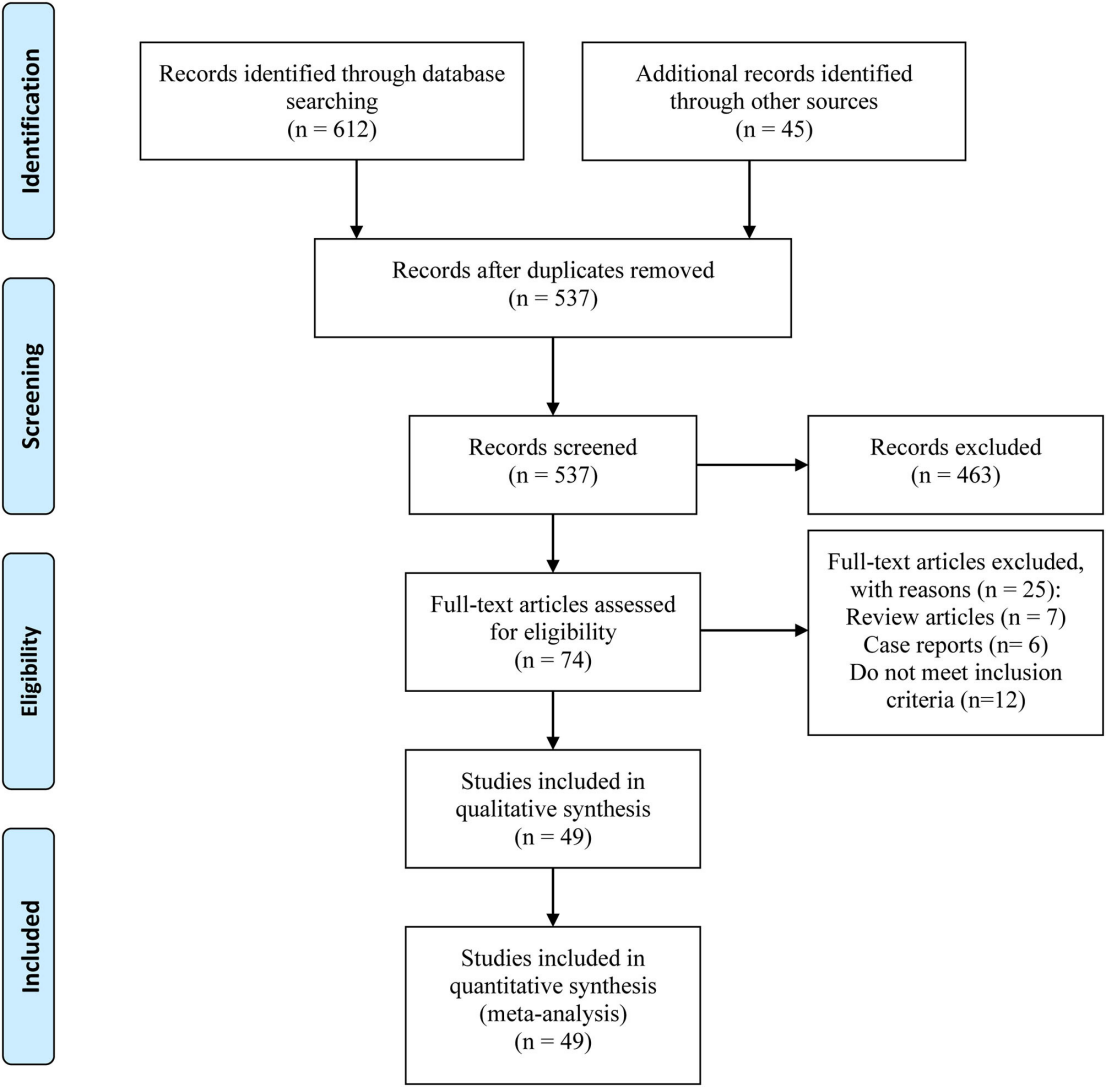
Flow diagram of the meta-analysis.

We included a total of 49 studies containing more than 10 million patients. The process of verifying the studies was in accordance with a PRISMA flow diagram (Figure 1). The basic characteristics are listed in Table 1. The studies were published between 1991 and 2019. A total of 38 studies were case-control studies (10, 11, 13, 19, 22–29, 31–35, 37, 39–43, 46–60) and 11 were retrospective cohort studies (9, 17, 18, 20, 21, 30, 36, 38, 44, 45, 61). About 12 of them were studied in the Asian populations, 24 in Europeans, and the remaining 13 in mixed ethnicities. Forty-one studies were graded higher than seven stars, while eight of them were six stars.

**Table 1:** Baseline information of the included studies.

**Included study**	**Country**	**Main ethnicity**	**Study duration**	**Study design**	**Match**	**Sample** **(** ***n*** **)**		**Gender (Male/** **Female**, ***n*****)**		**Mean/median** **age (years)**		**Quality score**	**Risk factors**
						**Case**	**Control**	**Case**	**Control**	**Case**	**Control**		
Chen et al. (17)	Chinese Taipei	Asian	2007–2013	Retrospective cohort study	NA	180	77,030	48,570/28,640		67.4 ± 12.2		******	13
Yang et al. (18)	Korea	Asian	2002–2013	Retrospective cohort study	Age, sex, systemic risk factors, co-medications-matched	28	10,081	8,075/2,034		NA		*******	8
Yang et al. (19)	Korea	Asian	2002–2013	Retrospective cohort study	Age, sex, systemic risk factors, co-medications-matched	1,097	399,877	523/574	176,163/183,358	NA		********	13
Lee et al. (20)	Korea	Asian	2011–2015	Case-control study	Age, sex, systemic risk factors-matched	22,498	31,475	9,175/13,323	12,832/ 18,643	58 ± 11	58 ± 11	*******	14
Sun et al. (21)	Chinese Taipei	Asian	1996–2013	Retrospective cohort study	Age, sex-matched	374	42,066	NA		NA		********	8
Chen et al. (22)	China	Asian	2013–2015	Case-control study	Age, sex-matched	71	142	47/24	94/48	54.86 ± 10.07	55.08 ± 10.36	*******	4, 6, 11
Zhu et al. (23)	China	Asian	2011–2016	Case-control study	Age, sex, laboratory parameters-matched	48	96	19/29	49/50	56.7 ± 11.9	57.3 ± 10.9	*******	16
Kuhli-Hattenbach et al. (24)	Germany	European	2010–2015	Case-control study	Age-matched	8	25	5/3	11/14	45.6 ± 7.2	42.9 ± 8.5	*******	2, 12
Flahavan et al. (25)	UK	European	2010–2015	Case-control study	NA	279	NA	279/0		61.8 (8.3)		*******	15
Kim et al. (26)	Korea	Asian	2009–2015	Case-control study	Age, gender-matched	45	45	19/26	19/26	63.48 ± 15.23	62.42 ± 12.51	*******	3, 4, 5, 6, 13
Zotz et al. (27)	Germany	European	1999–2005	Case-control study	Age, gender-matched	109	109	66/43	66/43	58.1 ± 11.1	57.2 ± 10.7	*******	5, 11, 12
Yao et al. (28)	China	Asian	2011–2015	Case-control study	Age, gender-matched	42	100	19/23	44/56	63.2 ± 3.6	63.4 ± 2.3	*******	14
Sahin et al. (29)	Turkey	European	2011–2015	Case-control study	Age, gender-matched	46	90	23/23	39/51	57.3 ± 9.1	55.7 ± 13.2	*******	4, 6, 13, 14
Lee et al. (30)	Chinese Taipei	Asian	2000–2011	Retrospective cohort study	Age, gender-matched	414	789	239/175	461/328	55.9 ± 18.6	55.1 ± 18.4	********	4, 5, 6, 7, 10
Chang et al. (31)	Chinese Taipei	Asian	2000–2009	Case-control study	Age, gender-matched	184	187,424	98/86	92,822/94,602	NA		********	4, 5, 6, 14
Cestari et al. (9)	USA	Mixed	2001–2014	Retrospective cohort study	NA	977	1,380,500	529/448	557761/ 822,739	64.0 ± 9.2	58.4 ± 9.4	******	1, 2, 5, 6, 7, 8, 13, 14
Nathoo et al. (32)	Canada	Mixed	2000–2011	Case-control study	Age-matched	1,109	1,237,290	1,109/0	1,237,290/0	69.8 ± 12.6	69.8 ± 12.6	******	5, 6, 7, 9, 10, 15
Campbell et al. (33)	Mixed	Mixed	2008–2012	Case-control study	Self control	43		43/0		61.4 (48–73)		*******	15
Sakai et al. (13)	Japan	Asian	NA	Case-control study	NA	34	102	20/14	67/36	62 (48–91)	64 (42–88)	*******	2, 3, 4, 5, 6, 11
Nagy et al. (19)	Hungary	European	2008–2012	Case-control study	NA	21	39	11/10	17/22	63.43 ± 10.78	73.07 ± 9.58	******	1, 2, 10, 14
Bilgin et al. (34)	Turkey	European	NA	Case-control study	Age, gender, systemic risk factors-matched	27	27	15/12	15/12	64.9 ± 7.86	63.7 ± 5.24	******	8
Arda et al. (35)	Turkey	European	2010–2012	Case-control study	Age, gender, systemic risk factors-matched	20	20	14/6	14/6	60.90 ± 8.14	61.15 ± 7.23	******	8
Stein et al. (36)	USA	Mixed	2001–2007	Retrospective cohort study	NA	3,123	2,257,163	NA		NA		*******	8
Markoula S (37)	Greece	European	2004–2007	Case-control study	Age, gender-matched	47	76	29/18	47/29	66.2	65.6	********	4, 5, 6, 11
Lee et al. (38)	USA	Mixed	1991–2007	Retrospective cohort study	NA	319	50,711	20,463/30,567		NA		********	2, 6
Felekis et al. (39)	Greece	European	2003–2008	Case-control study	Age, gender-matched	77	60	50/27	32/28	63.4 ± 9.3	66.3 ± 9.3	********	4, 5, 6, 11
Kuhli-Hattenbach et al. (40)	Germany	European	2004–2006	Case-control study	Age, gender-matched	35	70	27/8	52/18	51.5	49.8	********	12
Giambene et al. (41)	Italy	European	NA	Case-control study	Age, gender-matched	85	107	39/46	47/60	65 (26–88)	65 (21–84)	*******	3, 4, 5, 6, 11, 12,
Kesler et al. (42)	Israel	European	NA	Case-control study	Age, gender, smoking, atherothrombotic factors-matched	33	151	20/13	NA	62.5 ± 0.3	61.9 ± 0.1	*******	10
Pinna et al. (43)	Italy	European	1992–2006	Case-control study	Age, gender-matched	150	280	68/72	NA	63.6 ± 11.2	NA	*******	4, 5, 6, 7, 9
French et al. (44)	USA	Mixed	2004–2005	Retrospective cohort study	Age, smoking, systemic risk factors-matched	3,702,474		3,702,474/0		67		*******	15
						3,775,840		3,775,840/0		68			
Li et al. (10)	USA	Mixed	2000–2004	Case-control study	Age, gender-matched	73	73	38/35	38/35	63.5 ± 11.0	63.5 ± 11.1	********	3, 5, 6, 7, 8, 9, 13, 14
Margo and French (45)	USA	Mixed	2004–2005	Retrospective cohort study	NA	4,157,357		4,157,357/0		64		*******	15
Palombi et al. (46)	France	European	NA	Case-control study	Age, gender-matched	27	5,615	18/9	2,648/2,967	65	63.5	*******	8
Nagy et al. (47)	Hungary	European	1997–2004	Case-control study	Age, gender-matched	36	81	23/13	53/28	65.9 ± 11.6	61.6 ± 12.6	*******	3, 4, 5, 6, 7, 11, 12
Salomon (48)	Israel	European	1984–2000	Case-control study	NA	92	145	66/26	85/60	61.8 ± 11.7	57.3 ± 18.4	******	1, 2, 14
Glueck et al. (49)	USA	European	1999–2003	Case-control study	Age, gender, race-matched	12	36	4/8	12/24	62 ± 15	63 ± 15	*******	11
Deramo et al. (11)	USA	European	1995–1998	Case-control study	Age, gender-matched	37	74	25/12	50/24	43.2	43	*******	3, 4, 5, 6, 14
Weger et al. (50)	Austria	European	1996–2000	Case-control study	Age, gender-matched	71	71	30/41	30/41	68.1 ± 8.7	68.3 ± 9.4	*******	3, 4, 6, 7, 9, 14
Mojon et al. (51)	USA & Switherland	Mixed	9 months	Case-control study	Age, gender-matched	17	17	15/2	15/2	64.6 ± 11.7	63.3 ± 11.0	*******	3, 5, 6, 7, 8
Salomon et al. (52)	Israel	European	1984–1999	Case-control study	NA	74	71	54/20	43/28	64.5 (41–89)	66	******	2, 11
Pianka et al. (53)	Israel	European	1998–1999	Case-control study	Age, gender-matched	40	81	NA		66.3 ± 1.96	66 ± 2	*******	12
Salomon et al. (52)	Israel	European	1984–1997	Case-control study	Age, gender-matched	61	90	45/16	53/37	62 (34–85)	66 (31–85)	*******	3, 4, 5, 6, 7, 11,
Jacobson et al. (54)	USA	Mixed	1987–1995	Case-control study	Age, gender-matched	51	NA	30/21	NA	68 (48–86)	NA	********	3, 5, 6, 7, 14
Johnson et al. (55)	USA	European	1993–1996	Case-control study	Race, cup-disc ratio matched	43	30	27/16	14/16	70.7	77.9	*******	2, 3, 4, 6, 9, 14
Talks et al. (56)	UK	European	1986–1992	Case-control study	Age, gender-matched	41	41	27/14	27/14	66.7	66.9	*******	3, 4, 5, 6, 12, 14
Fry et al. (57)	USA	Mixed	1987–1990	Case-control study	Age, sex-matched	15	30	7/8	NA	61	61	*******	16
Kalenak et al. (58)	USA	Mixed	NA	Case-control study	Age, gender, race-matched	45	45	21/24	21/24	66.7 ± 9.1	66.6 ± 9.7	*******	5, 6, 7, 13, 14

*NA, Not available*.

*Risk factors: 1. age; 2. gender; 3. smoking; 4. hypertension; 5. hyperlipidemia; 6. DM; 7. CHD; 8. sleep apnea; 9. cerebrovascular disease; 10. cardiovascular drugs; 11. genetic factors; 12. hypercoagulation; 13. ocular risk factors; 14. other factors; 15. phosphodiesterase-5 inhibitors; 16. carotid stenosis*.

*represents score of Newcastle-Ottawa Scale (NOS).

**Figure 2:**
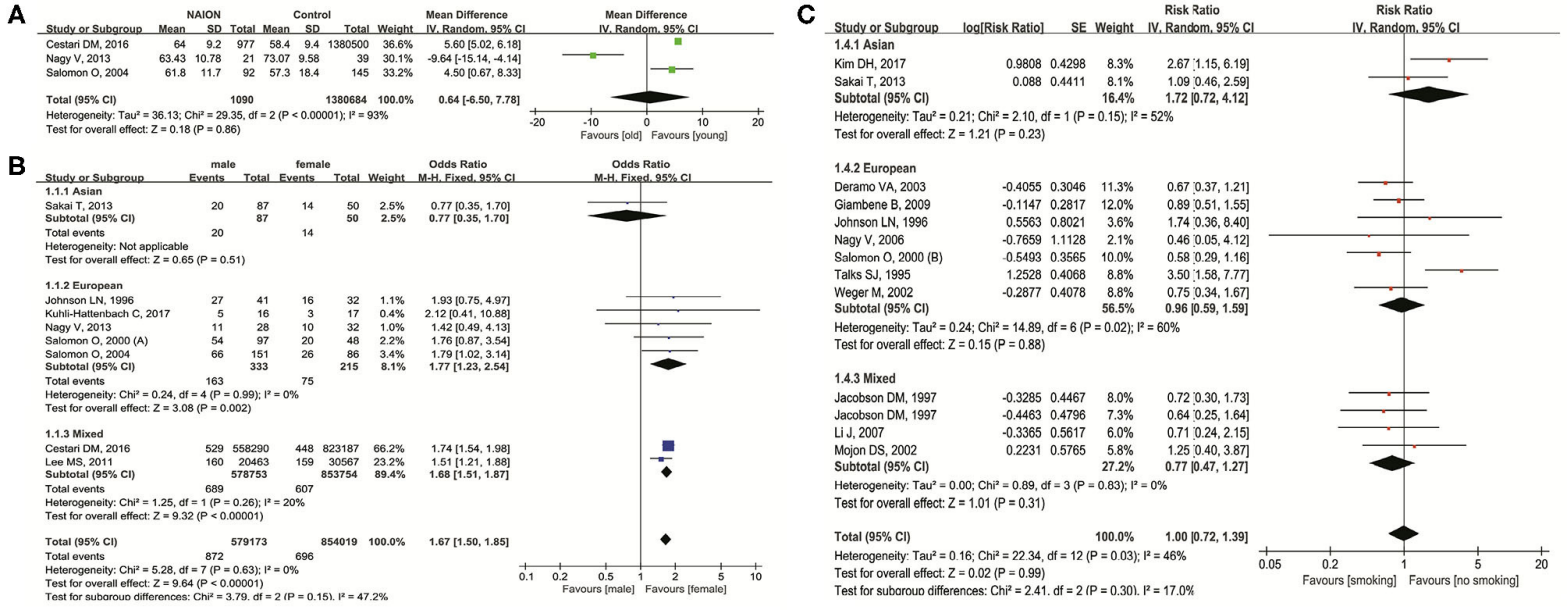
Meta-analysis of the association of nonarteritic anterior ischemic optic neuropathy (NAION) with age **(A)**, gender **(B)**, and smoking **(C)**.

Figure 2 shows the relationship between NAION and age, gender, and smoking status. In three studies (9, 19, 48) investigating the influence of age on NAION, results indicated no significance (MD = 0.64 years, 95% *CI*: −6.50 to 7.78, *P* = 0.86) with heterogeneity (*I*^2^ = 93%, *P* < 0.00001) (Figure 2A). In eight studies (9, 13, 19, 24, 38, 48, 52, 55) concerning gender, men had significant higher incidence of NAION than women (*OR* = 1.67, 95% *CI*: 1.50–1.85, *P* < 0.00001) without heterogeneity (*I*^2^ = 0%, *P* = 0.63). In the subgroups, five studies in Europeans also demonstrated the higher susceptibility in men (*OR* = 1.77, 95% *CI*: 1.23–2.54, *P* = 0.002). Two studies in mixed ethnicities reached similar results (*OR* = 1.68, 95% *CI*: 1.51–1.87, *P* < 0.00001) (Figure 2B). No heterogeneity existed in both the subgroups. One Asian study showed no difference in NAION incidence between men and women (*OR* = 0.77, 95% *CI*: 0.35–1.70, *P* = 0.51). There were no obvious publication biases detected through the inverted funnel plots of these factors.

Twelve studies (10, 11, 13, 26, 41, 47, 50, 51, 54–56, 59) researched the impact of current smoking status on NAION and found no significant association (*RR* = 1.00, 95% *CI*: 0.72–1.39, *P* = 0.99) with slight heterogeneity (*I*^2^ = 46%, *P* = 0.03). In the subgroup analysis based on the ethnicities, results in all the three groups (Asian, European, and mixed) pointed out no significant association (Figure 2C). The shape of the inverted funnel plot was approximately symmetrical and the Egger's test did not show any indication of publication bias (*t* = 0.41, *P* = 0.691).

**Figure 3:**
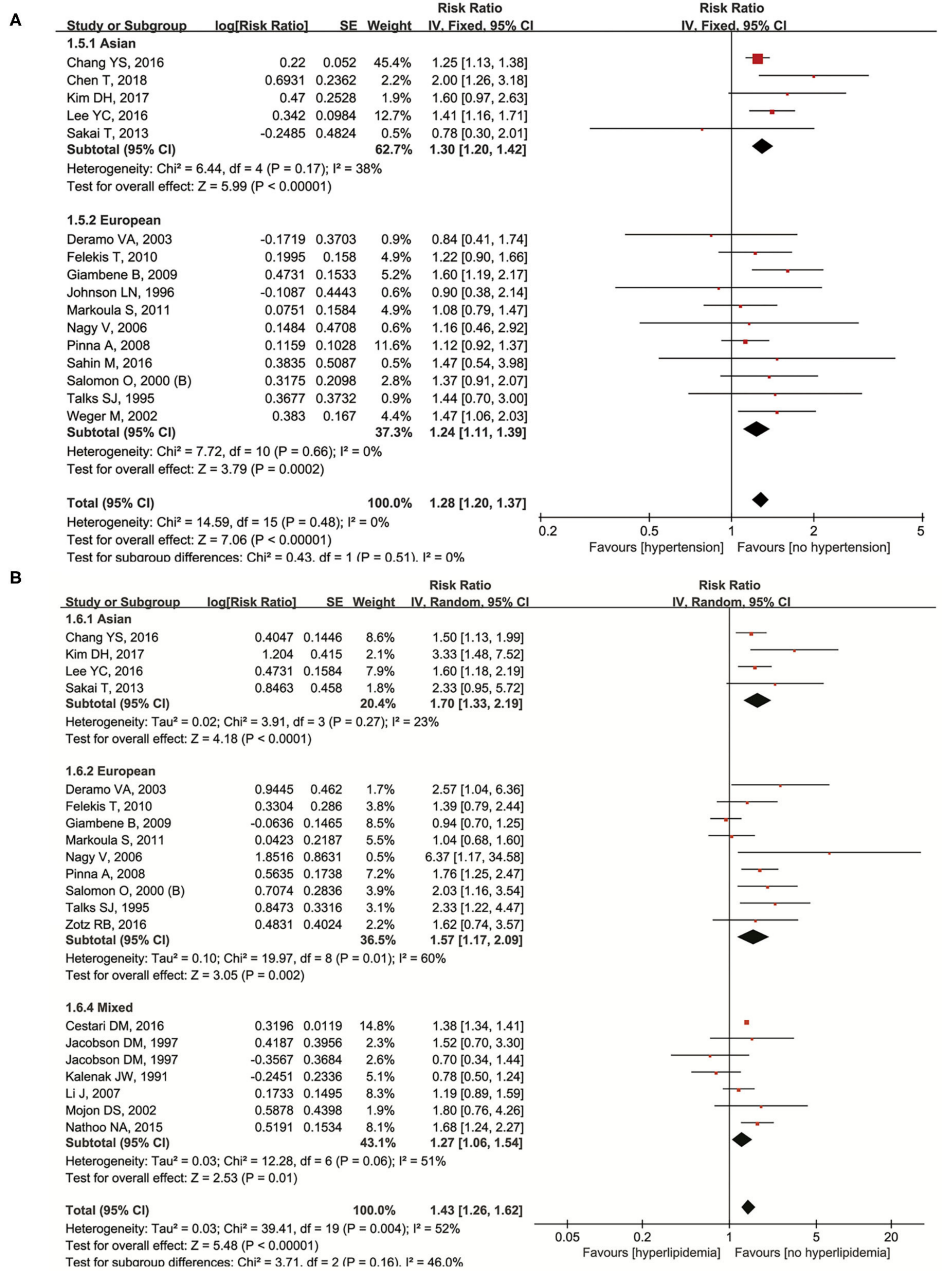
Meta-analysis of the association of NAION with hypertension **(A)** and hyperlipidemia **(B)**.

Hypertension was studied in the 16 original articles (11, 13, 22, 26, 29–31, 37, 39, 41, 43, 47, 50, 55, 56, 59). Our results showed that it was a significant risk factor of NAION (*RR* = 1.28, 95% *CI*: 1.20–1.37, *P* < 0.00001) with no heterogeneity (*I*^2^ = 0%, *P* = 0.48). In five of the studies on Asians, a significant higher incidence of NAION was found in hypertensive patients (*RR* = 1.30, 95% *CI*: 1.20–1.42, *P* < 0.00001) and 11 European studies achieved similar results (*RR* = 1.24, 95% *CI*: 1.11–1.39, *P* < 0.00001). Both the cases did not demonstrate heterogeneity (Figure 3A). No significant publication bias was detected through either the inverted funnel plot or the Egger's test (*t* = 0.24, *P* = 0.817).

We extracted and pooled data from the 19 original studies (9–11, 13, 26, 27, 30–32, 37, 39, 41, 43, 47, 51, 54, 56, 58, 59) researching hyperlipidemia. It was a significant risk factor of NAION (*RR* = 1.43, 95% *CI*: 1.26–1.62, *P* < 0.00001), and heterogeneity was detected (*I*^2^ = 52%, *P* = 0.004). According to the subgroup analysis, more NAION cases were found in the hyperlipidemia patients among the studies on Asian (*RR* = 1.65, 95% *CI*: 1.35–2.01, *P* < 0.00001), European (*RR* = 1.57, 95% *CI*: 1.17–2.09, *P* = 0.002), and mixed populations (*RR* = 1.27, 95% *CI*: 1.06–1.54, *P* < 0.01). Heterogeneity appeared in the European and mixed groups, while in Asians no heterogeneity was found (Figure 3B). Neither the inverted funnel plot nor the Egger's test showed publication bias (*t* = 0.97, *P* = 0.347).

**Figure 4:**
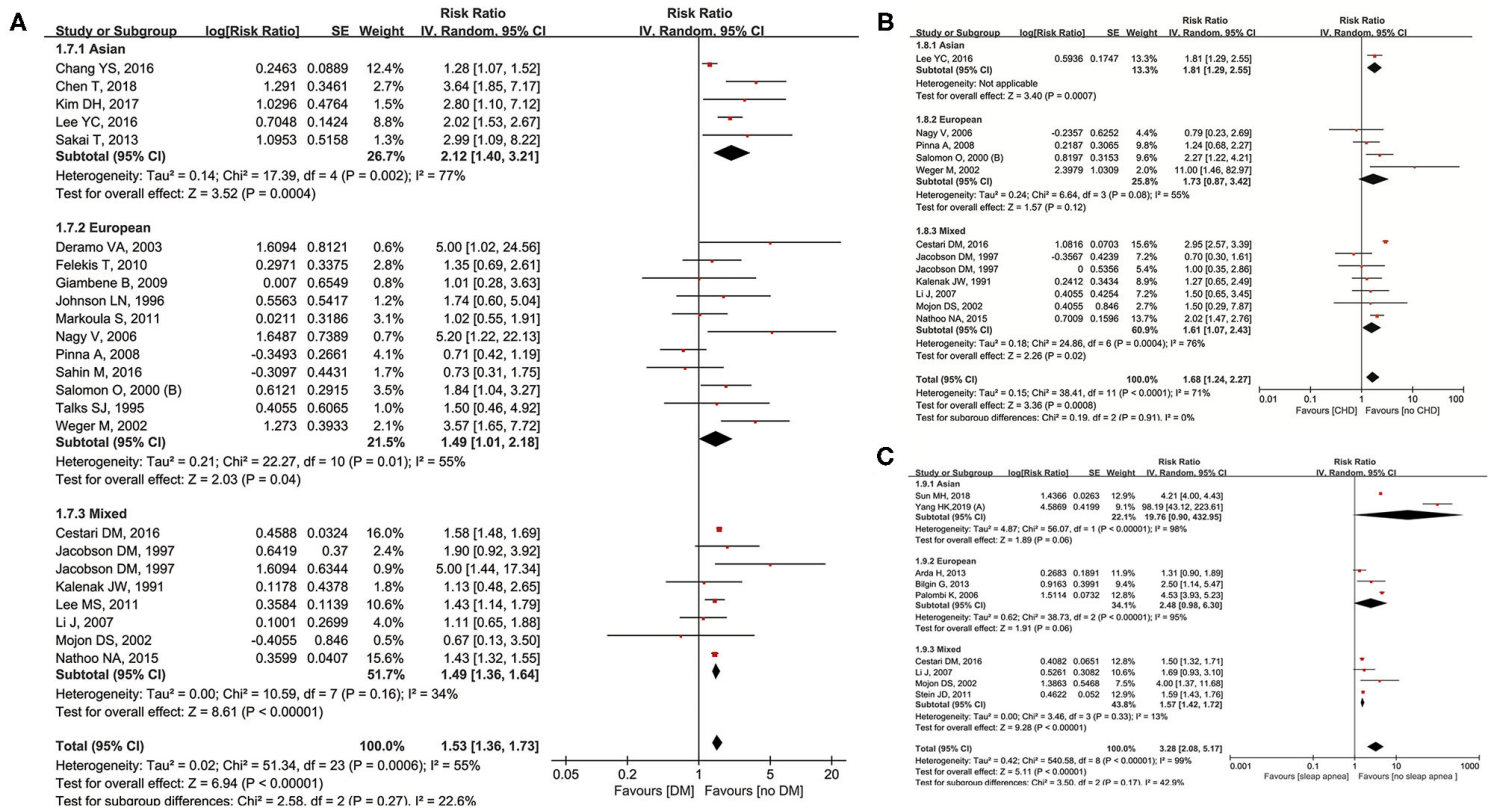
Meta-analysis of the association of NAION with diabetes mellitus (DM) **(A)**, coronary heart disease (CHD) **(B)**, and sleep apnea **(C)**.

Twenty-three studies (9–11, 13, 22, 26, 29–32, 37–39, 41, 43, 47, 50, 51, 54–56, 58, 59) were included for examining the relationship between DM and NAION. In our meta-analysis, diabetes significantly increased the occurrence of NAION (*RR* = 1.53, 95% *CI*: 1.36–1.73, *P* < 0.00001) and results had heterogeneity (*I*^2^ = 55%, *P* = 0.0006). The subgroup analysis was shown as below: *RR* was 2.12 (95% *CI*: 1.40–3.21, *P* = 0.0004) in Asians, 1.49 (95% *CI*: 1.01–2.18, *P* = 0.04) in Europeans, and 1.52 (95% *CI*: 1.44–1.59, *P* < 0.00001) in mixed populations. Heterogeneity was shown together with the forest plot (Figure 4A). The inverted funnel plot and the Egger's test showed no publication bias (*t* = 1.34, *P* = 0.203).

The data from 11 original studies (9, 10, 30, 32, 43, 47, 50, 51, 54, 58, 59) were collected for pooled results of coronary heart disease (CHD) on NAION. Our forest plot indicated CHD to be a significant risk factor with *RR* = 1.68 (95% *CI*: 1.24–2.27, *P* = 0.0008). Heterogeneity was also significant (*I*^2^ = 71%, *P* < 0.00001). In the subgroups, one Asian study (*RR* = 1.81, 95% *CI*: 1.29–2.55, *P* = 0.0007) and six studies on mixed ethnicities (*RR* = 1.61, 95% *CI*: 1.07–2.43, *P* = 0.02) found significant association, while no such relationship was detected in four of the Europeans studies (*RR* = 1.73, 95% *CI*: 0.87–3.42, *P* = 0.12). Heterogeneity was obvious in the European and mixed groups, while not applicable in the Asian group (Figure 4B). No significant publication bias was detected through either the inverted funnel plot or the Egger's test (*t* = −1.00, *P* = 0.346).

Nine studies (9, 10, 34–36, 38, 46, 51, 61) investigated sleep apnea and its correlation with NAION. Sleep apnea impacted the existence of NAION significantly (*RR* = 3.28, 95% *CI*: 2.08–5.17, *P* < 0.00001) bearing heterogeneity (*I*^2^ = 99%, *P* < 0.00001). Four studies on the mixed ethnicities observed positive association (*RR* = 1.57, 95% *CI*: 1.42–1.72, *P* < 0.00001) without heterogeneity (*I*^2^ = 13%, *P* = 0.33), while three of the European studies demonstrated no influence of sleep apnea on NAION (Figure 4C) in the subgroup analysis. The inverted funnel plot did not show publication bias.

**Figure 5:**
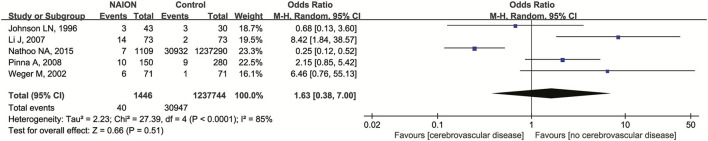
Meta-analysis of the association of NAION with cerebrovascular diseases.

Cerebrovascular disease was investigated in a total of five studies (10, 32, 43, 50, 55). No significant association was detected after we pooled the original data (*OR* = 1.63, 95% *CI*: 0.38–7.00, *P* = 0.51). Heterogeneity was found significantly (*I*^2^ = 85%, *P* < 0.00001) (Figure 5). No publication bias was detected through the inverted funnel plot.

The laboratory biochemical markers measuring coagulative status were examined and studied (27, 40, 41, 47, 53, 56). Our meta-analysis found the following three factors to be significantly related to NAION: increased fibrinogen (*RR* = 1.78, 95% *CI*: 1.26–2.52, *P* = 0.001), hyperhomocysteinemia (*RR* = 3.14, 95% *CI*: 1.99–4.93, *P* < 0.00001), and high level of lipoprotein(a) (*RR* = 1.36, 95% *CI*: 1.09–1.71, *P* = 0.007) (Table 2). All the results showed no heterogeneity. Elevated VIII factor had no significant influence on NAION (*RR* = 1.91, 95% *CI*: 0.79–4.63, *P* = 0.15) with apparent heterogeneity (*I*^2^ = 82%, *P* = 0.04). There were no publication biases detected through the inverted funnel plot for all the above factors.

**Table 2:** Meta-analysis results of the association of hypercoagulation, cardiovascular drugs, and ocular risk factors with non-arteritic anterior ischemic optic neuropathy (NAION).

**Factors**	**No. of studies**	**Sample size**		**Risk**		
		**Cases (*n*)**	**Controls (*n*)**	**Pooled OR/RR (95% CI)**		***P***
**Hypercoagulation**					
Increased fibrinogen	3	186	231	1.78 (1.26–2.52)		0.001
Hyperhomocysteinemia	4	269	367	3.14 (1.99–4.93)		<0.00001
Increased lipoprotein(a)	3	229	286	1.36 (1.09–1.71)		0.007
Elevated VIII factor	3	180	260	1.91 (0.79–4.63)		0.15
**Cardiovascular drugs**					
Antithrombotics	3	468	979	2.30 (1.86–2.84)		<0.00001
Statins	3	1,556	1,238,230	1.32 (1.18–1.48)		<0.00001
β-blockers	2	447	940	1.48 (1.05–2.08)		0.02
**Ocular factors**					
Microaneurysm/retinal hemorrhage	1	45	45	5.37 (1.09–26.49)		0.04
Small cup	1	45	45	35.20 (4.45–278.25)		0.007
Small cup to disc ratio	1	40	120	NA		<0.00001
IOP	1	46	90	1.27 (1.08–1.48)		0.003
AMD	1	977	1,380,500	3.29 (2.85–3.80)		<0.00001
RVO	1	977	1,380,500	9.72 (7.95–11.89)		<0.00001
Number of anti-VEGF injections	1	180	77,030	<10 vs. 10–15 times	1.91 (1.32–2.76)	0.0006
				<10 vs. >15 times	2.20 (1.42–3.43)	0.0004
Post-cataract surgery	1	1,097	399,877	1.80 (1.46–2.21)		<0.00001
Glaucoma	3	1,095	1,380,618	0.74 (0.11–5.00)		0.76

*IOP, intraocular pressure; AMD, age-related macular degeneration; RVO, retinal vein occlusion; OR, odds ratio; RR, rate ratio; VEGF, vascular endothelial growth factor*.

Data of cardiovascular drugs history were collected and pooled. Three studies (19, 30, 42) inspected the relationship of antithrombotics and NAION. Significant association was detected (*OR* = 2.30, 95% *CI*: 1.86–2.84, *P* < 0.00001) with no heterogeneity (*I*^2^ = 0%, *P* = 0.39). In detail, a significant increase in the NAION cases was found in patients taking statins (*OR* = 1.32, 95% *CI*: 1.18–1.48, *P* < 0.00001). Beta-blockers (β-blockers) were also investigated in the two studies with significant correlation to NAION (*OR* = 1.48, 95% *CI*: 1.05–2.08, *P* = 0.02) (Table 2).

We analyzed the six genotypes and gained pooled results from the nine included studies (13, 22, 27, 37, 39, 41, 47, 49, 59). Factor V Leiden heterozygous cases were more susceptible to NAION (*RR* = 2.21, 95% *CI*: 1.19–4.09, *P* = 0.01), while five genotypes were not significant: MTHFR 677T heterozygous (*OR* = 0.96, 95% *CI*: 0.11–8.40, *P* = 0.97), MTHFR 677T homozygous (*OR* = 1.78, 95% *CI*: 0.58–5.49, *P* = 0.31), ACE DD (*OR* = 0.92, 95% *CI*: 0.44–1.89, *P* = 0.81), ACE II (*OR* = 0.87, 95% *CI*: 0.59–1.30, *P* = 0.51), and ACE ID (*OR* = 1.09, 95% *CI*: 0.70–1.70, *P* = 0.69). Inverted funnel plots did not show the publication biases.

**Figure 6:**
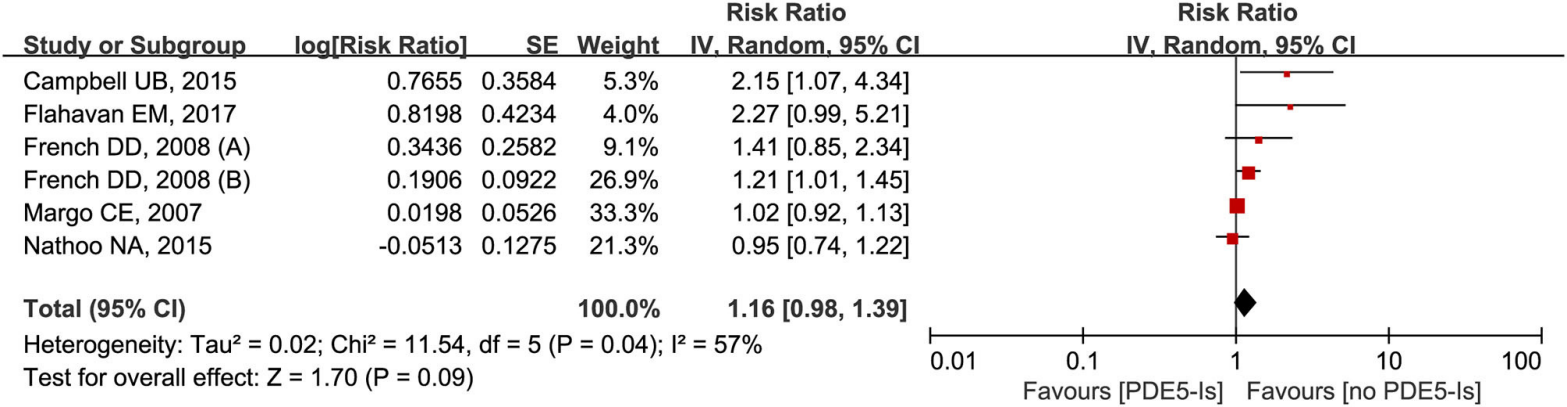
Meta-analysis of the association of NAION with the use of phosphodiesterase type-5 inhibitors (PDE5-Is) within 1-month.

Six trials from the five studies were analyzed for the impact of 1-month use of PDE5-Is on NAION (25, 32, 33, 44, 45). Results from meta-analysis showed no significant increased trends of NAION incidence (*RR* = 1.16, 95% *CI* = 0.98–1.39, *P* = 0.09) with obvious heterogeneity (*I*^2^ = 57%, *P* = 0.04) (Figure 6). No obvious publication bias was detected through the inverted funnel plot visual inspection.

Apart from the systematic diseases, ocular factors were also studied and recorded comprehensively in the published literature; however, they were mentioned in less than the three articles. In three studies (9, 10, 58) concerning glaucoma, no significant association was found (*OR* = 0.74, 95% *CI*: 0.11–5.00, *P* = 0.76) with heterogeneity (*I*^2^ = 90%, *P* < 0.00001). Microaneurysm/retinal hemorrhage, small optic cup, high intraocular pressure (29), small cup to disc ratio (CDR) (60), age-related macular degeneration, retinal vein occlusion (9), anti-vascular endothelial growth factor injection times (17), and post-cataract surgery (18) were all studied in one literature, which is listed in Table 2.

Table 3 provides 14 factors researched in <3 studies. Positive B allele of the glycoprotein Ibα variable number of tandem repeats (48), increased P-selectin (19), decreased flow-mediated dilation (28), anemia (9), peripheral vascular occlusive disease (9, 58), recurrent herpes labialis (55), increased mean platelet volume (24), elevated IgG titer to Chlamydia pneumonia (50), and end-stage renal disease (31), all indicated a significant association with NAION, while depression (9), COPD (10, 54), hypothyroidism (11, 56), ischemic kidney injury (38), and carotid stenosis (23, 57) had no such relationship.

**Table 3:** Meta-analysis results of the association of rare factors with NAION.

**Factors**	**No. of studies**	**Sample size (** ***n*** **)**		**Risk**	
		**Case**	**Control**	**Pooled OR/RR (95% CI)**	***P***
VNTR B allele	1	92	145	4.25 (1.67–10.82)	0.002
P-selectin	1	NA		4.12 (1.22–13.91)	0.02
Flow-mediated dilation	1	NA		1.79 (1.67–2.01)	<0.00001
Anemia	1	977	1,380,500	2.13 (1.83–2.47)	<0.00001
Depression	1	977	1,380,500	0.87 (0.72–1.06)	0.17
Peripheral vascular occlusive disease	2	1,022	1,380,545	1.99 (1.52–2.59)	<0.00001
Recurrent herpes labialis	1	43	30	3.11 (1.18–8.19)	0.02
Mean platelet volume	1	46	90	1.61 (1.13–2.28)	0.008
IgG titer to Chlamydia pneumoniae	1	71	61	3.48 (1.26–9.61)	0.02
COPD	3	73	73	0.80 (0.34–1.85)	0.6
Hypothyroidism	2	78	115	2.68 (0.61–11.72)	0.19
End-stage renal disease	1	184	187,424	2.60 (1.88–3.59)	<0.00001
Ischemic kidney injury	1	22,498	31,475	1.53 (0.67–3.46)	0.31
Carotid stenosis	2	63	126	39.70 (0.02, 99269.34)	0.36

*VNTR, a variable number of tandem repeats; COPD, chronic obstructive pulmonary disease; OR, odds ratio; RR, rate ratio; CI, confidence interval; NA, not available*.

## Discussion

In our systematic review and meta-analysis, we included articles studying a variety of risk factors: age, gender, ethnicity, systematic diseases, ocular factors, genotypes, cardiovascular drugs, and so on. We finally concluded the following risk factors to be significantly associated with NAION: male gender, hypertension, hyperlipidemia, DM, CHD, sleep apnea, medication history of cardiovascular drugs, and factor V Leiden heterozygous. Some other systematic and ocular diseases were researched in <3 studies and did not seem to be significant risk factors. In the subgroup analyses based on ethnicities, we found that the influences of gender in Asians and CHD and sleep apnea in Europeans were not significant. Therefore, we should take notice when applying our results to different populations.

In our meta-analysis, the cardiovascular factors were highly associated with NAION and studied in most literature, including hypertension, hyperlipidemia, and DM. NAION probably results from topical and/or systematic hypoperfusion (2). Although this is not a thrombotic event, many predisposing factors related to thrombogenesis or hypercoagulative state can disturb the systematic blood circulation *via* different pathways (41). For example, hyperlipidemia is harmful to endothelial cells and accelerates the formation of atherosclerotic plaques, further leading to hypertension and CHD (27). Some biochemical markers (such as hyperhomocysteinemia) and genetic polymorphisms (such as factor V Leiden heterozygous) indicating hypercoagulative state were also significant risk factors in our meta-analysis. Therefore, the above factors are co-factors of NAION with similar mechanisms. Drugs treating cardiovascular diseases were proved to be significantly associated with NAION, too, such as antithrombotics, β-blockers, and statins. These drugs could not be considered as independent risk factors because they were only applied to treat the diseases.

Although the above major factors were reported to induce NAION, we summarized and reconfirmed these conclusions. In addition, we included the hypercoagulative biomarkers and possible risky genetic polymorphisms from the published literature. We first performed the meta-analyses on these factors, demonstrating that increased homocysteinemia, fibrinogen, lipoprotein(a), and factor V Leiden heterozygous were risk factors of NAION. Several diseases and biomarkers were identified in <3 studies, which are listed in Table 3. They might be the potential risk factors if more original studies were carried out. Specifically, we conducted the subgroup analyses based on ethnicities in seven risk factors. The cases were divided into Asians, Europeans, and mixed ethnicities from original publications. The ethnicity did not affect the association between smoking, hypertension, hyperlipidemia, DM, and NAION. Nevertheless, the incidence of NAION had no disparity in different gender among the Asians. CHD and sleep apnea were not significant risk factors in Europeans. This subgroup analysis expanded the application of our results to different peoples.

Diabetes and sleep apnea were proved to be important risk factors in the previously published meta-analyses (7, 14). We reconducted meta-analyses on both factors with 11 new studies included on diabetes and three on sleep apnea. Apart from the verification of their conclusions with larger sample sizes, we also performed the subgroup analyses according to ethnicities as stated above. For DM, its influence on NAION was not related to ethnicity in our pooled results, similar to the conclusions of Chen et al. (14). Furthermore, our conclusions were more powerful with three extra cohort studies compared with the previous meta-analysis (14), in which the included studies were all case-controlled because the cohort studies had a higher level of evidence. Sleep apnea was not a significant risk factor of NAION in the Europeans after our subgroup analysis, although a published study found that the Europeans were more likely to have NAION (14). However, only three studies were included in this subgroup, and we achieved marginal data (*P* = 0.06). We expected more valid original studies on different ethnicities, in case, a high-quality meta-analysis could be conducted.

We found no apparent association between the occurrence of NAION and 1-month use of PDE5-Is, which was published by us in 2018 (8). Although PDE5-Is mainly cause vasodilation and systematic hypotension (32), their influences on the NAION pathogenesis remain controversial. Because of the relatively low incidence of NAION and difficulties in diagnosis, it was hard to include adequate samples in the published literature, and confounders were not adjusted in several case-control studies. More clinical studies are necessarily needed to provide strong evidence on this point.

A crowded optic disc was often observed in NAION because hypoperfusion or ischemia of the optic nerve head is apparent in a tight optic disc structure (26). An optical coherence tomography (OCT) is a useful tool to measure the CDR. Several studies showed the association between the CDR and NAION. For example, González Martín-Moro et al. reported a smaller CDR to be a risk factor and poor prognostic marker of NAION based on the measurements by OCT (60). OCT angiography can detect the perfusion and vessel density of optic disc in patients with NAION, further providing more information of blood supply around optic nerve head. Fard et al. and Gandhi et al. found that the patients with NAION lost some vascular networks and perfusion in the optic disc areas (62, 63). However, most of the studies compared the CDR between NAION and controls, but did not explain the definition of “small optic disc.” Pooled *OR*s or *RR*s of small CDR on NAION could not be extracted from these studies, so they did not meet our inclusion criteria. A meta-analysis studying this topic can be conducted in our future work, specifically collecting the continuous variables from these original articles.

Some other factors were researched in <3 studies. Johnson and colleagues (55) researched the impact of recurrent herpes labialis on NAION and reached a significant relationship. Weger et al. observed an elevated IgG titer to Chlamydia pneumonia in NAION cases compared with normal controls (50). Both pathogens disturb the function of endothelial cells, activate the secretion of inflammatory cytokines, and thus induce or promote atherosclerosis. In the cohort study by Chang et al., end-stage renal disease was proved to increase the risk of NAION (31), probably because these patients had received repeated hemodialysis, which resulted in systematic hypotension. Zhu et al. (23) and Fry et al. (57) achieved different results on the relationship between carotid stenosis and NAION. Although the carotid arterial abnormities can possibly cause ocular arterial disturbance by potential embolism and decreased perfusion (64), no consistent conclusions were reached due to the lack of well-planned studies with large sample sizes. All the above research were carried out in a small number of studies, so the studies with larger samples size are necessary to confirm these findings.

Although our meta-analysis was conducted comprehensively and included multiple potential risk factors of NAION, it still has several limitations. First, all the included original articles were case-control or retrospective cohort studies. No RCTs or prospective cohort studies have been published yet. Recalled data might be incomplete and inaccurate, bringing biases to these studies. Second, most NAION possible factors have similar mechanisms and act as confounders. Independence of these factors may probably be examined by RCTs; however, no RCTs can be conducted on studying the risk factors. Third, the time span of the included studies lasts from 1991 to 2019, during which the changes in lifestyles and medical techniques influence the type of risk factors. Finally, some risk factors were examined in <3 studies and it was hard to confirm their association with NAION. The above limitations reduced the quality of our meta-analysis partly and restricted our results to be applied.

Consequently, our study concluded that the following risk factors were associated with NAION: male gender, hypertension, hyperlipidemia, DM, CHD, sleep apnea, medication history of cardiovascular drugs, and factor V Leiden heterozygous. Better understanding of these risk factors in NAION can direct future research and therapeutic approaches.

## Data Availability Statement

The original contributions presented in the study are included in the article/supplementary material, further inquiries can be directed to the corresponding author/s.

## Author Contributions

BL, TD, and DX: conceptualization and project administration. BL and YY: literature search and screening, formal analysis, data curation, and writing—original draft preparation. BL and TD: methodology and software. BL, YY, and TD: validation. WL, TD, and DX: writing—review and editing. TD and DX: supervision. All authors contributed to the article and approved the submitted version.

## Funding

This study was supported by a grant from the Natural Science Foundation of Guangdong Province, China (No. 2020A1515110113) and the Science and Technology Planning Project of Guangzhou, China (No. 202102020113).

## Conflict of Interest

The authors declare that the research was conducted in the absence of any commercial or financial relationships that could be construed as a potential conflict of interest.

## Publisher's Note

All claims expressed in this article are solely those of the authors and do not necessarily represent those of their affiliated organizations, or those of the publisher, the editors and the reviewers. Any product that may be evaluated in this article, or claim that may be made by its manufacturer, is not guaranteed or endorsed by the publisher.
